# New insights in the Spanish gene pool of olive (*Olea europaea* L.) preserved *ex situ* and *in situ* based on high-throughput molecular markers

**DOI:** 10.3389/fpls.2023.1267601

**Published:** 2024-01-05

**Authors:** Francisco Jesús Gómez-Gálvez, Antònia Ninot, Juan Cano Rodríguez, Sergio Paz Compañ, Javier Ugarte Andreva, Javier Alfonso García Rubio, Isis Pinilla Aragón, Javier Viñuales-Andreu, José Casanova-Gascón, Zlatko Šatović, Ignacio Jesús Lorite, Raúl De la Rosa-Navarro, Angjelina Belaj

**Affiliations:** ^1^ Mejora Vegetal y Biotecnología, Instituto Andaluz de Investigación y Formación Agraria, Pesquera, Alimentaria y de la Producción Ecológica (IFAPA), Centro Alameda del Obispo, Córdoba, Spain; ^2^ Fruticultura, Institut de Recerca i Tecnologia Agroalimentàries (IRTA), Mas Bové, Constantí, Tarragona, Spain; ^3^ Ingeniería y Tecnología Agroalimentaria, Instituto Andaluz de Investigación y Formación Agraria, Pesquera, Alimentaria y de la Producción Ecológica (IFAPA), Centro Venta del Llano, Mengíbar, Jaén, Spain; ^4^ Olivicultura, Instituto Valenciano de Investigaciones Agrarias (IVIA), Moncada, Valencia, Spain; ^5^ Servicio de Investigación Agraria y Sanidad Vegetal, Gobierno de La Rioja, Logroño, Spain; ^6^ Universidad de Zaragoza, Escuela Politécnica Superior de Huesca, Aragón, Spain; ^7^ Department of Plant Biodiversity, Faculty of Agriculture, University of Zagreb, Zagreb, Croatia; ^8^ Centre of Excellence for Biodiversity and Molecular Plant Breeding (CroP-BioDiv), Zagreb, Croatia; ^9^ Department of Plant Breeding, Institute for Sustainable Agriculture, Spanish National Research Council (IAS-CSIC), Cordoba, Spain

**Keywords:** genetic characterization, *Olea europaea*, EST-SNPs, cultivars collection, conservation

## Abstract

In Spain, several local studies have highlighted the likely presence of unknown olive cultivars distinct from the approximately 260 ones previously described in the literature. Furthermore, recent advancements in identification techniques have significantly enhanced in terms of efficacy and precision. This scenario motivated a new nationwide prospecting effort aimed at recovering and characterizing new cultivated germplasm using high-throughput molecular markers. In the present study, the use of 96 EST-SNP markers allowed the identification of a considerable amount of new material (173 new genotypes) coming from areas with low intensification of production in different regions of Spain. As a result, the number of distinct national genotypes documented in the World Olive Germplasm Bank of IFAPA, Córdoba (WOGBC-ESP046) increased to 427. Likewise, 65 and 24 new synonymy and homonymy cases were identified, respectively. This rise in the number of different national cultivars allowed to deepen the knowledge about the underlying genetic structure. The great genetic variability of Spanish germplasm was confirmed, and a new hot spot of diversity was identified in the northern regions of La Rioja and Aragon. Analysis of the genetic structure showed a clear separation between the germplasm of southern and northern-northeastern Spain and indicated a significantly higher level of admixture in the latter. Given the expansion of modern olive cultivation with only a few cultivars, this cryptic germplasm is in great danger of disappearing. This underlines the fact that maintaining as many cultivars as possible will increase the genetic variability of the olive gene pool to meet the future challenges of olive cultivation.

## 1 Introduction

The olive tree (*Olea europaea* L.) is one of the quintessential emblems of Spain, constituting a crucial element in the economic, social and environmental framework of the country. Its cultivation in Spain could dates back to the Bronze Age (Terral and Arnold-Simard, 1996; Terral et al., 2004), although Phoenicians, Greeks and especially Romans and Muslims were the main responsible for its expansion and cultivation by importing know-how and plant material from eastern Mediterranean (Kaniewski et al., 2012; Besnard et al., 2018). Thus, most of the cultivated genotypes of olive tree in Spain have their origin in the empirical selection made by farmers over the centuries, being most of them very old and confined to its presumed area of selection (Barranco, 2010). Besides, the number of cultivars obtained by olive breeding is very small compared to other fruit trees.

Nowadays, Spain is by far the country with the largest number of olive trees planted in the world and represents the leading olive-producing country with more than 20% of the world area and around 34% of the worldwide production (FAOSTAT, 2020). One of the reasons that have led to the achievement of this leading position is the modernization and technification of olive farming carried out during the last decades (Fernandez-Escobar et al., 2013). These changes have been linked to a certain reconversion in the cultivar landscape. A broad range of old, local, and traditional cultivars are being displaced by few cultivars that are well known for their desirable traits in terms of earliness of bearing, oil content and quality, and suitability for new harvesting and pruning techniques (de la Rosa et al., 2007). As an example, Spanish olive-growing areas are dominated by only three cultivars: ‘Arbequina’, ‘Picual’ and ‘Hojiblanca’ (Belaj et al., 2016). Moreover, these few cultivars represent the main source of supply for most of the national breeding programs, thus favouring even more a possible genetic erosion of the crop (Rallo et al., 2013; León et al., 2021; Yilmaz-Duzyaman et al., 2022). This genetic erosion poses a risk to the legacy of diversity built over generations of olive growers and compromises the added value of exclusivity provided by local cultivars in olive products. Moreover, this loss of diversity weakens the availability of a potentially valuable strategic reserve for breeders that could help for dealing with future challenges such as temperature increase (potentially leading to a lack of chilling requirements for flowering and/or heat stress), water stress, salinity, emerging pests and diseases, farming in new edaphoclimatic areas, and new market trends such as the search for specific quality characters in EVOO (Pérez et al., 2019; Medina-Alonso et al., 2020; Serrano et al., 2020; Lorite et al., 2022). For these reasons, the recovery, conservation and study of minor cultivars is of increasing interest in Spain and elsewhere (Díez et al., 2011; Hmmam et al., 2018; Ninot et al., 2018; Debbabi et al., 2020; Valeri et al., 2022; Marchese et al., 2023).

To address this need, the World Olive Germplasm Bank of Córdoba (WOGBC) plays a crucial role by conserving as much olive genetic patrimony as possible. WOGBC is established at the experimental field “Alameda del Obispo” of the Andalusian Institute for Research and Training in Agriculture, Fishery, Food and Organic Production (IFAPA), and represents a reference olive germplasm bank both at national (INIA-ESP046) and international level (IOC) (Belaj et al., 2016; Díez et al., 2018; Belaj et al., 2022). The accurate identification of olive material is a crucial task in germplasm banks (Atienza et al., 2013; Trujillo et al., 2014; El Bakkali et al., 2019). Historically, different morphological and molecular markers, especially simple sequence repeats (SSRs), have been used at WOGBC (Barranco et al., 2000; Belaj et al., 2012; Atienza et al., 2013; Trujillo et al., 2014). Currently, cultivar identification in WOGBC is performed by means of EST-SNP markers (Single-Nucleotide Polymorphism from Expressed Sequence Tags). EST-SNP markers have shown clear advantages over previously used markers: fully automation in high-throughput assays, cost-effective, lower genotyping error rates, and higher reproducibility across different laboratories, germplasm collections, and genotyping platforms (Belaj et al., 2018). In a recent research aimed at improving the management and use of the genetic resources maintained at WOGBC, a core set of 96 EST-SNP markers was evaluated for the fingerprinting of 1273 accessions from 29 countries. It allowed the accurate identification of the highest number of olive genotypes (668) up to date, that are currently maintained at the WOGBC collection. Among them, 38% belonged to Spanish cultivars (Belaj et al., 2022). Most of these Spanish cultivars were incorporated to the collection thanks to the prospecting surveys conducted at the end of the past century (Barranco, 2010). Since then, several identification studies that explored different areas of the country at local level have shown evidences of uncatalogued cultivars that remained to be recover and preserve (Íñiguez et al., 2001; Viñuales-Andreu, 2007; Díez et al., 2011; Fernández i Martí et al., 2015; Ninot et al., 2018). These findings indicate that the real number of Spanish olive cultivars is still underestimated and point out the importance of continuous and systematic prospecting surveys.

Starting from this scenario, in which a precisely identified set of national material is available and a management protocol has been fairly refined, the enrichment of the WOGBC with national unknown cultivars has been seen as a must in the last years. Thus, a new wave of prospecting and collecting surveys on Spanish territory was deployed to recover this local untapped diversity. The present research is part of an ongoing project aimed at enriching WOGBC by introducing new local Spanish cultivated germplasm followed by its genetic and agronomic characterization. In particular, this work addresses: i) the search for and recovery of non-catalogued cultivars, ii) their fingerprinting and identification by means of 96 EST-SNP markers, and iii) the assessment of their genetic diversity and structure.

## 2 Material and methods

### 2.1 Plant material

**Figure 1:**
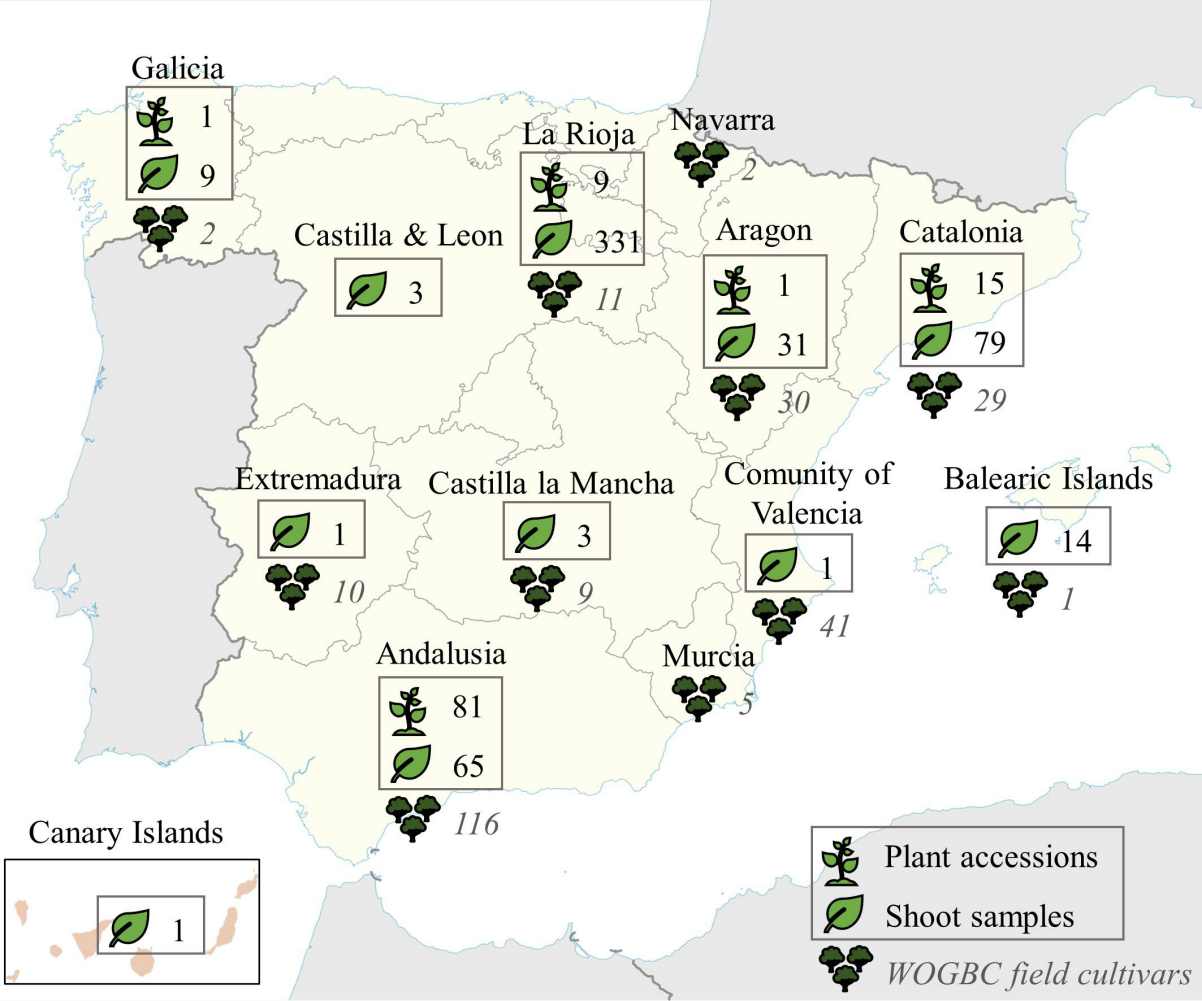
Geographical distribution of the new plant material acquired recently and subjected to identification in the present study (in boxes), and geographical origin of the national cultivars already conserved ex situ in the WOGBC field.

The collection of plant material under study was made in two principal ways: 1) through collaborations and incorporation of plant accessions from regional collections such as the Institut de Recerca i Tecnologia Agroalimentaria, IRTA, (Catalonia, Northeastern Spain), and the Servicio de Investigación Agraria y Sanidad Vegetal (La Rioja, Northern Spain); and 2) through ongoing local prospecting surveys conducted mainly in Aragon, La Rioja and Catalonia (Northern, Northeastern Spain) and Andalusia (Southern Spain) regions and at a minor scale in other regions of the country (**Table 1**, **Figure 1**, **Supplementary Table 1**). In the case of Andalusia, special focus was placed in prospecting uncatalogued cultivars previously identified by Díez et al. (2011) from monumental and centennial trees (trunk diameter ranging 1-2.72 m) as well as other minor local cultivars that escaped from previous surveys; as for Aragon and Catalonia, most prospecting material came from the mountainous system of Pre-Pyrenees region. In order to reduce redundancies, sampling collection was implemented following the strategy defined in Belaj et al. (2022). In this sense, as much characterization data as possible was collected to be included in the passport data (fruit and stone size and shape, and any other relevant agronomical information), and an *a priori* identification by means molecular markers was conducted before their introduction into WOGBC collection. For this identification, the genetic profiles of all the samples included in this study were compared among them and with the WOGBC database (see Data analysis section below). The *in situ* morphological and agronomical information of sampled trees served to ensure their cultivated status. The *a priori* identification was made from shoot samples collected from olive trees conserved *in situ*, which were georeferenciated. However, when sampling involved vulnerable trees at risk of disappearance and/or growing in very remote areas with difficult access, they were vegetatively propagated, and further incorporated as new accessions at different propagation facilities of WOGBC. Thus, *a priori* identification was conducted in a total of 538 DNA samples obtained from plant shoots of olive trees surveyed in different sites *in situ* (**Table 1**, **Figure 1**, **Supplementary Table 1**) as well as in 107 new accessions recently incorporated to the propagation facilities of the WOGBC for their *ex situ* conservation (each accession composed of 1 to 4 olive plants).

**Table 1:** Number of samples and accessions genotyped per region and number of different cultivars identified.

Type of plant material	Sampling sites/origin	Number of samples/accessions	Genotypes identified	New Genotypes*
**DNA from plant shoots (collected *in situ*)**	Andalusia	65	43	25
	Aragon	31	20	7
	Catalonia	79	68	34
	La Rioja	331	76	35
	Other Regions	32	27	14
	**TOTAL**	**538**	234 (215**)	**115**
**WOGBC accessions in propagation facilities (collected *ex situ*)**	Andalusia	81	54	32
	Aragon	1	1	1
	Catalonia	15	15	15
	La Rioja	9	9	9
	Galicia	1	1	1
	**TOTAL**	**107**	**80**	**58**

*New genotypes whose profile did not match that of any other sample of this study or any of the profiles included in the WOGBC database.

**Total genotypes omitting redundancies between regions: 15 genotypes were identified in two different regions, and two of them (‘Picual’ and ‘Manzanilla de Sevilla’) were identified in three diferent regions (i.e., 19 redundancies).

### 2.2 EST-SNP genotyping

DNA was extracted from fresh leaves and sprouts following de la Rosa et al. (2002) and quantitatively and qualitatively evaluated using spectrophotometry (Nanodrop 2000, Thermo Scientific, Wilmington, DE). Genotyping was conducted in the Sequencing and Genotyping Unit of the University of the Basque Country, by means of a core set of 96 EST-SNPs and following the Fluidigm method as in Belaj et al. (2022). Briefly, two preamplification primers (Locus-Specific Primer (LSP) and Specific Target Amplification (STA) primer) amplified the target region containing the SNP to be genotyped. All 96 SNPs were preamplified simultaneously in one multiplex PCR, for each sample separately, on a Veriti Thermal Cycler (Applied Biosystems by ThermoFisher, Waltham, MA, USA). Afterwards, an additional PCR amplified a portion of the target SNP region, using the LSP and two fluorescently labelled allele-specific internal primers ASP1 and ASP2, containing either the first or the second allele, respectively. The second PCR was performed on a Fluidigm 96.96 Dynamic Array IFC (Integrated Fluidic Circuit), where reactions were performed in separate nano-wells for each SNP and sample combination, allowing simultaneous genotyping of 94 samples (+2 negative test controls - NTCs) at 96 SNP loci. This PCR was performed on a BioMark HD System (Fluidigm, South San Francisco, CA, USA) Finally, SNP genotypes were then determined by measuring the fluorescence intensity of both alleles normalised with respect to NTCs values, using SNP Genotyping Analysis Software (Fluidigm, South San Francisco, CA, USA). Two reference cultivars (‘Picual’ and ‘Frantoio’) were included in all PCR reactions as controls. Only samples with less than eight EST-SNP missing data were included for further analysis.

### 2.3 Data analysis

For the identification analysis of the plant material under study, the genetic profiles of all the samples included in this study were compared among them and with the WOGBC database that includes 668 different cultivars from around the world, 254 of them Spanish (Belaj et al., 2022). In addition, EST-SNP genotyping data were checked with passport data as well as *in situ* morphological and agronomical data from prospecting and collecting sites. Both the shoot samples and the new accessions maintained at different propagation facilities were considered as redundant or duplicates when they shared the same EST-SNP profiles within them and/or with WOGBC cultivars. For each redundancy group, a representative profile was selected, and the rest of redundant samples were excluded for further analysis.

For further diversity and population structure analysis, the genetic profiles of all the non-redundant Spanish genotypes identified were considered, i.e. including both the different genotypes newly identified together with the rest of national cultivars previously identified and maintained at the WOGBC (254 different genotypes; Belaj et al. (2022)) (**Figure 1**; **Supplementary Table 1**).

Key genetic parameters were calculated for the set of 96 EST-SNPs genotyped in the whole set of non-redundant Spanish genotypes, as well as for *a priori* groups defined according to their regional origin. The genetic differentiation between groups was calculated by AMOVA and fixation index (F_ST_) GenAlex 6.5 (Peakall and Smouse, 2012) and Cervus (Marshall et al., 1998) software were used to calculate the diversity parameters.

Population structure was first explored using the Bayesian-based approach implemented in the software package STRUCTURE v.2.2.4 (Pritchard et al., 2000) following the settings described in Belaj et al. (2022). The non-redundant Spanish genotypes were assigned to a specific cluster if their value of the corresponding Q-value (i.e., proportion of membership) were higher than 0.80, otherwise they were considered mosaic/admixed.

Discriminant Analysis of Principal Components (DAPC) was implemented as complementary clustering method to further explore the pattern of population structure (Jombart et al., 2010). In contrast to STRUCTURE, the DAPC is a multivariate method that uses a non-hierarchical approach for defining genetic clusters. The DAPC was performed in R 4.2.2 (R Core Team, 2021) using wrapper functions of the R package SambaR (de Jong et al., 2021) (https://github.com/mennodejong1986/SambaR). Since a preselected set of 96 markers was used (Belaj et al., 2022) and data were curated right after genotyping (genotypes with less than 8 missing data retained), the ‘filterdata’ function, mandatory when following the SambaR workflow, was run with settings that allowed to retain all EST-SNPs and samples under study. Functions ‘find.clusters’ and ‘dapc’ were run inside the pipeline of SambaR, and the number of principal components, clusters, and discriminant functions were considered according to Jombart and Collins (2015) tutorial.

## 3 Results

### 3.1 Genotyping

The EST-SNP genotyping revealed a considerable level of redundant germplasm within the plant material prospected and collected in the present study. About 60% of the 538 DNA samples taken from olive tree shoots conserved *in situ* shared the same EST-SNP profile with at least another collected sample, being identified 215 different genotypes among them (**Table 1**).In the case of the 107 olive accessions maintained ex situ, only ~25% showed redundancy among them, with 80 different genotypes identified. Additional redundant germplasm was found when expanding the comparison with the WOGBC EST-SNP database (668 different cultivars, 254 of them Spanish). Thus, 46.5% of the 215 different genotypes identified *in situ*, and 27.5% of the 80 different genotypes identified ex situ were found to be redundant with cultivars already maintained at WOGBC.

It is worth mentioning that all the redundancies were detected among cultivars previously identified in the Spanish territory, although some of them also presented synonymies with cultivars from other Mediterranean countries. The only exception was found in the sample ‘Olivo de Mallorca-2’, identified *in situ*, that matched with the French cultivar ‘Aglandau’.

The EST-SNP genotyping of the plant material under study, enabled the identification of new synonymy cases (identical fingerprints but different naming). Thus, a total of 65 new synonymy cases belonging to 39 different genotypes were identified. Most of the new synonymies belonged to cultivars already found in the WOGBC collection. However, seven new synonymy groups were identified for the first time in the present study and were detected among the new plant material included through collection or prospecting surveys (**Table 2**).

**Table 2:** New synonymy cases detected in the present study.

New synonymies detected	Representative cultivar*
Verdial	Acebuche de Autol
Manzanilla Castúa, Redondilla de Cuevas del Becerro	Alameño de Cabra
Arnellidero, Bermejuela, Serranilla	Arroniz
Aceituno, Calahorrana	Bodoquera
Picudillo, Silvestre	Bolvino
Vidrial de Cuevas	Buidiego
**Cabacenca**	**Llei del Bessó**
Cerruda de Artasona	Cerruda de Olvena
Grossal del Pallars, Grossal de Cadaqués	Safrawi (SYR); syn of spanish cv Cirujal
Negral	Cirujal de Préjano
Llargueta	Corbella
Olivo de los Pozos	Corralones de Andujar
Alcarreño	Changlot Real
Solimar	Farga
Millarenca	Gorda Limocillo
Aceituno, Pla	Gordal Sevillana
Campiñesa, Coloraillo de Cortijo Nuevo	Hojiblanca
Minuera	Lechín de Granada
Plans	Lloma
Casta Cortijuelos	Loaime
**Manzanal de Ráfales**	**Mançanal d’Arnes**
**Santa Lucia**	**Manzanella del Mezquin**
Menya de Vila-rodona	Menya
Verdial de Setenil	Morona
Marons	Morona de Castellon
Bonany, Carrasquenya, Torres Mil·leni	Morruda de Segorbe
Mas de Bot	Morrut
**Aceitunero, Pardo, Poncho, Redondilla, Sevillano**	**Negral de Préjano**
Corraleña	Negrillo de Arjona
Acebuche de la Hoya, Olivo de Vilares	Olivo de Los Prados
Sevillí	Palomar
Aceitunero, Vidrial, Cirujal	Picalaceña de Cornago
**Aceitunero, Negrillas**	**Picudillo**
Picalaceña, Navarrillo, Racimuda, Coloradillo	Redondilla de Logroño
**Rojal de Cabacés**	**Verdal de Bovera**
Carrasqueña, Machona, Macho, Machazo, Pardo, Tempranillo	Royal de Calatayud
**Desmayo**	**Verdal d’Arnes**
Vera del Vallès	Verdal de Manresa
Casta Dilareña	Zorzaleño de Granada

*Representative cultivars are selected according to historical identification and passport data at WOGBC collection. The new synonymy groups identified in this study for the first time are indicated in bold, with representative cultivars chosen according to higher occurrence or relevant information obtained when collecting.

Despite the high number of redundant accessions found in this study, a high number of new cultivars was identified. Thus, more than half of the genotypes identified within the DNA samples (115 out of 215) as well as more than 70% of genotypes identified within the new accessions (58 out of 80) were found to be different to any of the WOGBC cultivars. In spite of their local distribution, most of these new genotypes have been identified in more than one collecting site or in different trees within the same orchard, thus evidencing a conscious vegetative propagation by farmers, that is considered a hallmark of cultivated olive germplasm. Besides, unique genotypes (i.e. identified only at one collecting site and/or tree) with passport data evidencing likely cultivated status (large fruits and stones, rough stone surfaces, high productivity, regular planting density, etc.) could be cultivars for which evidences of clonal propagation are yet to be found. For instance, a centennial tree sampled in 2016 in Canary Islands shared the same EST-SNP-genotype with another tree prospected 5 years later in an abandoned olive orchard in the south-east of Andalusia. Overall, a total of 173 new and distinct genotypes have been identified in the present study and will be progressively incorporated to the collection. This increases up to 427 the number of national Spanish genotypes identified to date (**Supplementary Table 1**). When considering the prospecting areas, the regions of Andalusia, Catalonia and La Rioja were the ones where the highest number of new local cultivars were identified (57, 49 and 44, respectively). In the case of Andalusia, 33 of the new cultivars were obtained from a total of 65 centennial trees surveyed (data not shown). Finally, up to 24 homonymy groups (i.e., a common denomination referring to different cultivars) were detected, being five of them reported for the first time in the present work: “Casta/Castizo”, “Colorado/Coloradillo”, “Llei”, “Cerruda”, and “Vidrial”. The denomination based on the greenish colour of fruits (“Verde”, “Verdal”, “Verdial”, etc) was found to include 16 different cultivars (**Supplementary Table 2**).

### 3.2 Genetic diversity analysis

The 96 EST-SNP markers showed a relatively wide diversity in the 427 distinct Spanish genotypes under study (**Supplementary Table 3**). Minor allele frequency (MAF) values ranged from 0.192 to 0.498, with an average value of 0.376, and the proportion of markers with MAF <0.3 and >0.3 accounted for 20.8% and 79.2%, respectively. Shannon’s information index (I) values ranged from 0.49 to 0.69, with the mean value of 0.65. The observed heterozygosity (H_O_) values ranged from 0.30 to 0.79, averaging 0.53, whereas the mean expected heterozygosity (H_E_) was 0.46, ranging from 0.31 to 0.50. All but five EST-SNPs showed polymorphic information content (PIC) values over 0.30.

The genetic diversity was also estimated for groups defined *a priori* according to their regional origin or sampling sites (**Table 3**). The H_O_ and H_E_ ranged from 0.45 to 0.59, and from 0.41 to 0.46, respectively, depending on the region of origin. The group of genotypes from Balearic Islands showed more similarity between H_O_ and H_E_, reporting therefore the highest fixation index (**Table 3**). According to the one-way AMOVA, the region of origin explained a low percentage of variance (4%), although *φ_ST_* values among regions were significant (p ≤ 0.001; **Table 4**). The EST-SNP pairwise differentiation among Spanish olive cultivars at regional level showed that the ones from northern regions of Aragon and La Rioja were the most genetically differentiated from the rest (**Table 5**); the highest differentiation values were observed between genotypes from Aragon and Extremadura. Interestingly, the Andalusian genotypes showed high similarities with those sampled in various regions such as Murcia, Extremadura, Castilla La Mancha, Galicia and Balearic Islands.

**Table 3:** Summary of genetic diversity parameters estimated for the 427 different Spanish-genotypes grouped by region of origin*.

		N	Na	Ne	I	H_O_	H_E_	uH_E_	F
**Origin**	**Andalusia**	173	2.00	1.84	0.64	0.56	0.45	0.45	-0.24
	**Aragon**	39	1.99	1.73	0.59	0.48	0.41	0.41	-0.16
	**Balearic Islands**	11	2.00	1.87	0.65	0.45	0.46	0.48	0.03
	**Catalonia**	77	2.00	1.83	0.64	0.49	0.45	0.45	-0.10
	**Castilla La Mancha**	11	1.98	1.76	0.60	0.58	0.42	0.44	-0.37
	**Community of Valencia**	41	2.00	1.85	0.65	0.52	0.45	0.46	-0.13
	**Extremadura**	10	1.98	1.79	0.62	0.59	0.43	0.45	-0.35
	**Galicia**	5	1.93	1.73	0.57	0.51	0.40	0.44	-0.25
	**La Rioja**	55	2.00	1.72	0.59	0.51	0.40	0.41	-0.25
	**Murcia**	5	2.00	1.77	0.61	0.57	0.42	0.47	-0.33
	Total	427	2.00	1.85	0.65	0.53	0.46	0.46	-0.15

*N, Number of distinct genotypes; Na, Average number of observed alleles; Ne, Number of effective alleles; I, Shannon’s Information Index; H_O_, Observed Heterozygosity; H_E_, Expected Heterozygosity; uH_E_, Unbiased Expected Heterozygosity; F, Fixation Index.

**Table 4:** Analysis of molecular variance for 427 Spanish olive genotypes grouped by region of origin*.

Source	df	SS	MS	Est. Var.	%	*φ_ST_*	*P_(φ)_*
**Among Pops defined by origin**	9	787	87.4	0.960	4%	0.043	0.001
**Within Indiv**	427	10756	25.2	25.2	96%		

*df, degree of freedom, SS, sum of squares, MS, mean squares, Est. var., estimate of variance, %, Percentage of total variation, φST, Phi statistic, P(ϕ) φST probability level after 999 permutations.

**Table 5:** EST-SNP pairwise differentiation among Spanish olive cultivars at regional level*.

	Andalusia	Aragon	Balearic Islands	Catalonia	Castilla La Mancha	Community of Valencia	Extremadura	Galicia	La Rioja	Murcia
**Andalusia**	–	0.001	0.069	0.001	0.235	0.002	0.281	0.240	0.001	0.348
**Aragon**	**0.071**	–	0.006	0.007	0.004	0.001	0.001	0.012	0.027	0.038
**Balearic Islands**	0.022	**0.050**	–	0.014	0.033	0.010	0.024	0.022	0.008	0.388
**Catalonia**	**0.051**	**0.020**	0.091	–	0.003	0.006	0.002	0.028	0.001	0.123
**Castilla La Mancha**	0.005	**0.076**	0.073	**0.066**	–	0.023	0.233	0.181	0.007	0.171
**Community of Valencia**	**0.025**	**0.042**	0.167	**0.017**	**0.038**	–	0.029	0.135	0.001	0.296
**Extremadura**	0.002	**0.102**	0.103	**0.069**	0.010	**0.036**	–	0.191	0.004	0.214
**Galicia**	0.010	**0.086**	0.202	**0.052**	0.031	0.022	0.030	–	0.024	0.384
**La Rioja**	**0.059**	**0.018**	**0.059**	**0.043**	**0.054**	**0.050**	**0.087**	**0.087**	–	0.035
**Murcia**	0.002	**0.055**	0.000	0.023	0.034	0.004	0.024	0.000	**0.061**	–

*Values of FST are given below the diagonal (bold indicates significant differences), and corrected P-values are given above the diagonal.

### 3.3 Population structure

The highest ΔK value (332,23) detected by STRUCTURE software was for K = 3, while the second-best solution was K = 2 (ΔK = 266.52) (**Supplementary Figure 1**). The proportion of membership of each individual in each gene cluster was calculated (**Supplementary Table 1**; **Supplementary Figure 2**). At K = 3, the cluster A was predominant mostly in Northern (La Rioja) and North-eastern (Aragon and Catalonia) accessions, being most of them assigned with high membership values. Thus, 87 accessions were assigned to this cluster with Q ≥ 0.8, and among them, the cultivar ‘Negral de Bierge’, displayed the highest values of membership (98.5%). The cluster B was found in some accessions from Catalonia (North-East), Andalusia (South), and the Eastern regions of Valencia and Balearic Islands, being the cluster with the lowest number of genotypes with Q ≥ 0.8. The third cluster, C, was mainly represented by olive genotypes from Andalusia; i.e, 121 out of 155 genotypes with Q ≥ 0.8 were Andalusian. Besides, olive accessions from the regions of Galicia (North-west), Extremadura (West), Castilla la Mancha (Center) and Murcia (South-east), were also assigned to this cluster. In general, very high values of membership were found for the accessions assigned to this cluster, being the well-known cultivar ‘Hojiblanca’ its highest representative (Q = 0.97). Finally, a large number of genotypes (n = 174; about 41% of the total) showed membership values lower than Q ≥ 0.8 in any of the three clusters. Within the set of genotypes newly identified in this study, 53 genotypes were assigned to cluster A, 10 to cluster B, 35 to cluster C, and 75 remained as intermixed genotypes with Q < 0.80. The regions that showed a higher prevalence of admixture were Catalonia and, especially, the Community of Valencia, with 75% and 82% of their genotypes showing intermix.

**Figure 2:**
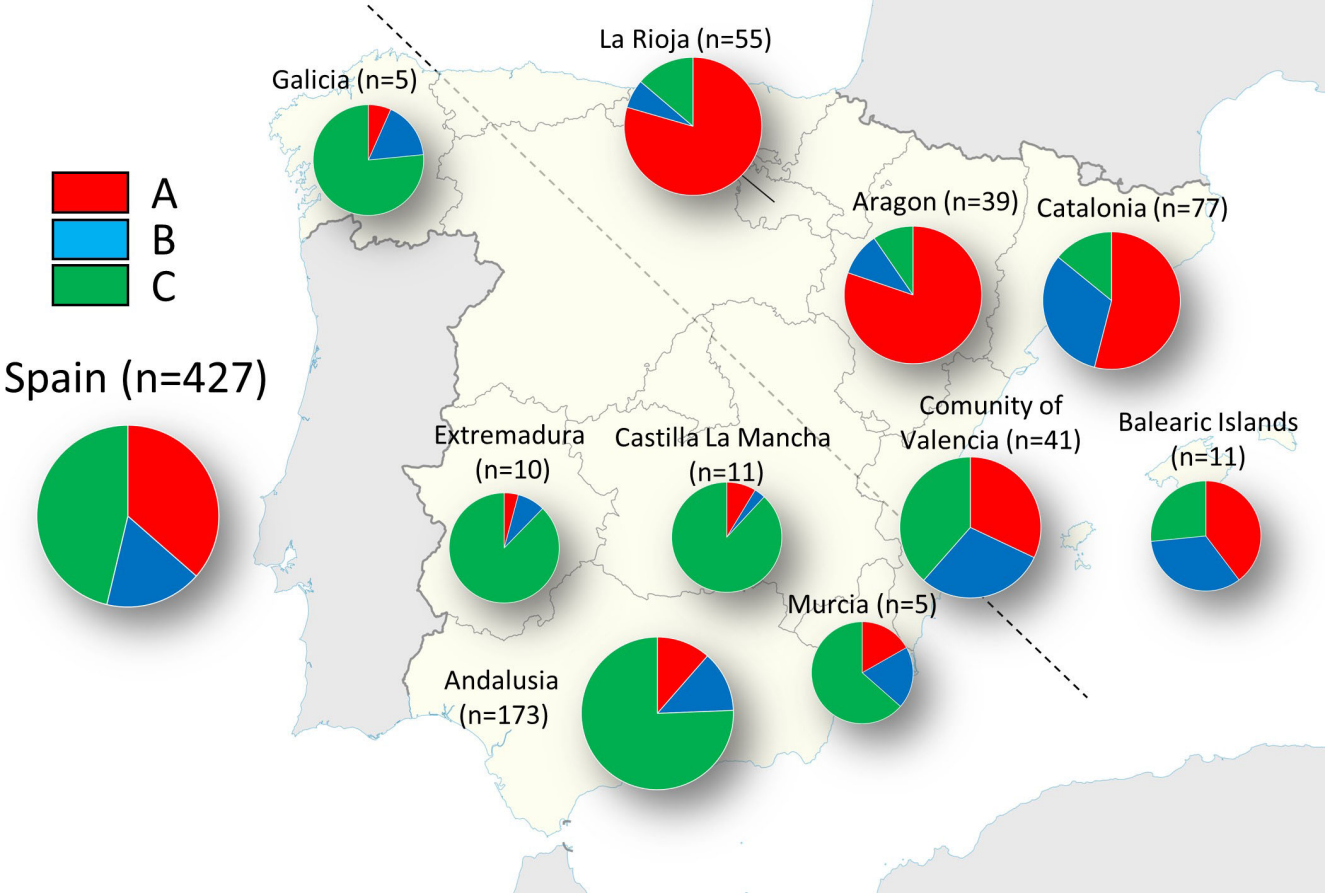
Pie charts representing the STRUCTURE clusters averaged by region for the 427 distinct genotypes.

STRUCTURE analysis revealed a certain geographic clustering of the Spanish olive accessions under study (**Supplementary Figure 2**, **Supplementary Table 1**). Such tendency could be seen clearly when the membership coefficient inferred for each cluster was averaged by region of origin (**Figure 2**). In this sense, a separation of Northern and North-eastern accessions (mainly assigned to cluster A) from the Southern, South-eastern, Central, Western and North-western accessions (mainly assigned to cluster C) could be discerned. Likewise, the Community of Valencia and the Balearic Islands, positioned in the intermediate zone, were the regions that showed more admixture, with a proportional distribution in the 3 clusters.

**Figure 3:**
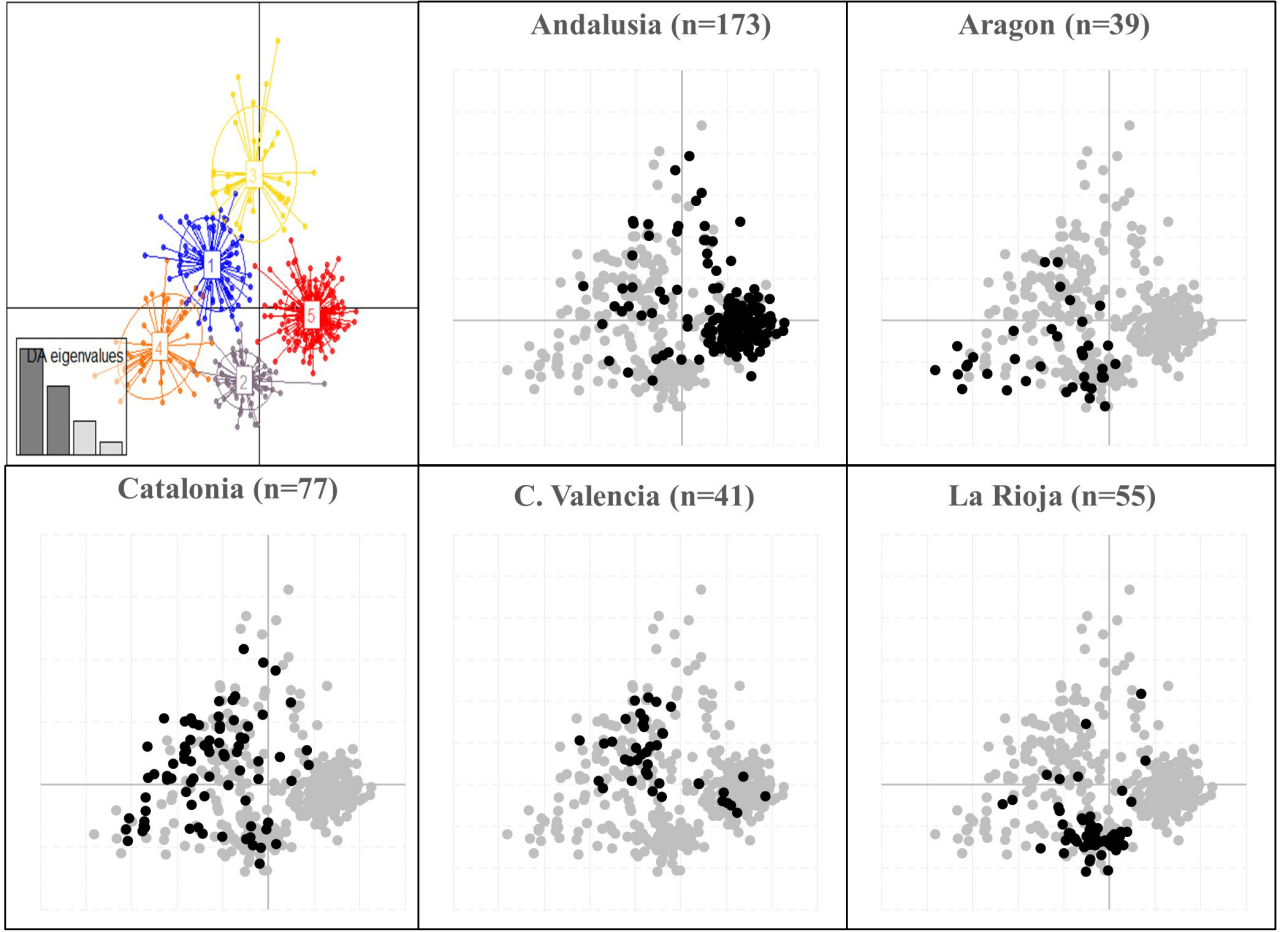
Scatter-plot of the discriminant analysis of principal components (DAPC) on a set of 427 Spanish olive genotypes identified by means 96 EST-SNP markers. Numbers and colors represent the five genetic groups found by the K-means method (see Jombart et al., 2010 for details). Regional differentiation is depicted in detail for those regions with n>30 genotypes.

The DAPC analysis performed without prior information on the accessions, identified five most likely genetic groups as indicated by BIC value (**Supplementary Figure 3A**). The first four Linear Discriminants functions and the first 80 Principal Components were retained for the analysis, representing more than 90% of total variability (**Supplementary Figure 3B, C**). Among the five groups identified, Group 1 included mainly genotypes from North-eastern (Catalonia, 44%) and Eastern (Valencia, 30.5%) regions; Group 2 comprised most of the genotypes sampled in Northern and North-eastern Spain, mainly in La Rioja (55%), Aragon (20.5%) and Catalonia (13%). Group 3 was represented by the lowest number of genotypes (38) and comprised mainly Andalusian (47.4%) and Catalonian (26.3%) genotypes. Group 4 consisted mainly of genotypes from Catalonia (42%) and Aragon (29%), while group 5 comprised mainly genotypes coming from the southern region of Andalusia (87%) (**Figure 3**; **Supplementary Table 1**). Thus, in total agreement with STRUCTURE, DAPC analysis revealed a clear differentiation between the genotypes mainly assigned to cluster A (Group 1, 2, 4) from the ones predominant to cluster B (Group 3) and C (Group 5).

## 4 Discussion

In the present study, the use of appropriate strategies for exploring, incorporation and management of olive genetic resources by means of EST-SNP markers together with an intensive collaborative network, made possible the collection and identification of 173 new Spanish cultivars. It is important to highlight that most of the new germplasm identified belongs to local and unknown cultivars. In this regard, in agreement with recent studies performed in olive (Hmmam et al., 2018; Debbabi et al., 2020; Atrouz et al., 2021; Valeri et al., 2022), the identification of a high number of local cultivars indicates that the olive crop still has a high local genetic variability that needs to be recovered before its disappearance. The ongoing incorporation of this untapped local diversity into WOGBC will contribute to fulfil its main goal, that is, to acquire, maintain, document, assess and make available as much genetic diversity of the crop as possible (Belaj et al., 2016; Belaj et al., 2022).

It is expected that chances of preserving and finding untapped diversity in olive is higher in those areas with less pressure of cultivar turnover and productivity (Belaj et al., 2022). In this sense, most of the new cultivars identified in northern areas (especially, Catalonia, La Rioja and Aragon) were probably neglected in previous national surveys, thus remaining uncatalogued throughout time, up to date. It is noteworthy the case of La Rioja, a region mainly known for its wine production, which, as shown here, has also a wide range of olive cultivars that have yet to be catalogued. In the case of Andalusia, in accordance with previous studies by means of SSR markers (Díez et al., 2011), an important number of the new cultivars identified were found in ancient trees growing in remote areas with complex topography and low productivity, i.e., areas with a low pressure of cultivar turnover. Other works that genetically characterized ancient olives trees in Mediterranean countries like Cyprus (Anestiadou et al., 2017), Israel (Barazani et al., 2014), Italy (Baldoni et al., 2006; Erre et al., 2010; Marchese et al., 2023), Malta (Valeri et al., 2022), Montenegro (Lazović et al., 2016), or Morocco (El Bakkali et al., 2013), also reported that only a small proportion of them matched to known olive cultivars. In agreement with the conclusion of these authors, our findings support that the ancient olives trees deserve a careful consideration and conservation measures as *in-situ* reservoir of olive genetic diversity.

The efficient identification of redundant germplasm prior to its introduction into a collection is as important as verifying and safeguarding as much diversity as possible. And it certainly contributes to an efficient management of olive genetic resources. In this regard, as already seen in previous studies in olive (Díez et al., 2011; Atienza et al., 2013; Lazović et al., 2016; Ninot et al., 2018; Belaj et al., 2022), our result indicate that prospecting surveys may constitute a gauge of redundant genotypes in the same or close olive growing areas, at both local and regional scale. In addition, EST-SNP genotyping of the plant material under study made possible the detection of 65 new synonymies that were not recorded so far. In this sense, our findings reinforce the need of *a priori* identification of the new plant material prospected to avoid the inclusion of duplicates into *ex situ* germplasm collections contributing thus to their cost-effective management.

Besides, this work enabled the identification of five new homonymy groups, as well as the enlargement of well-known homonymy groups with new members. For example, “Manzanilla” denomination, which refers to “apple fruit shape” and which constitutes the greatest group of homonymies documented in Spain (Barranco et al., 2005; Belaj et al., 2022), was enlarged with 7 additional, phenotypically different, cultivars: ‘Mançanal d’Arnes’(=‘Manzanal de Ráfales’), ‘Mançanenca d’Albagés’, ‘Mançanenca de Batea’, ‘Manzanella del Mezquín’, ‘Manzanilla de Alfarnatejo’, ‘Manzanilla Baquetera’, ‘Manzanilla Castúa’ (=‘Alameño de Cabra’).

Although various diversity studies have been conducted on olive germplasm at the national and regional level in Spain (Belaj et al., 2010; Díez et al., 2011; Trujillo et al., 2014; Fernández i Martí et al., 2015; Ninot et al., 2018), the present study constitutes the largest one performed with such a large number of Spanish genotypes and using EST-SNPs markers. The genetic variability displayed by the set of 96 EST-SNPs on the 427 nonredundant Spanish genotypes was very similar to that obtained when using the same set of markers on 668 nonredundant Mediterranean genotypes maintained in the WOGBC (Belaj et al., 2022). This confirms the wide diversity of cultivated olive germplasm in Spain.

When evaluating the genetic variability by region, it was observed that genotypes from the northern ones were the most genetically different with respect to the rest of the Spanish regions. This could possibly indicate local adaptation of these genotypes to colder and wetter local environmental conditions (some genotypes in the pre-Pyrenees were localized above 800 m.a.s.l.) than those found in the rest of Spain (Fernández i Martí et al., 2015).

In total accordance, STRUCTURE and DAPC analysis revealed a certain geographic clustering of Spanish cultivars. Thus, the olive accessions under study clustered in three main gene pools, being the ones from North-North-eastern and Southern Spanish provinces, the most clearly differentiated. This regional differentiation is in agreement with previous studies conducted with other molecular markers in olive (Sanz-Cortés et al., 2001; Belaj et al., 2010; Díez et al., 2015; Jiménez-Ruiz et al., 2020). Some authors have suggested that this separation might be due to different routes of expansion of olive growing from the Eastern Mediterranean Basin along the South and the North coasts (Jiménez-Ruiz et al., 2020; Julca et al., 2020). In addition, a possible local selection, specifically adapted to particular environmental conditions and fulfilling agronomic expectations, may explain some of the differences found between northern and southern Spanish olive accessions. Besides, human displacement of olive cultivars under harsh agroclimatic events, might have shaped the spread and diversification of the olive tree as documented in historic literature. The Andalusian agronomist Al-Tignari documented an exuberant importation of olive trees brought in ships from northern Africa to repopulate the Al-Andalus olive grove devastated by a long drought occurred at the end of the Visigothic kingdom (mid-6th to early 8th century) (Guzmán Álvarez, 2004; Guzman Álvarez, 2007; Orlandis, 2011). Also, there are some notes about frosts and disease outbreaks in different regions of Spain that served as an incentive for olive growers to replant, renew or abandon their main cultivars during the 19th and 20th centuries (Guzman Álvarez, 2007). Finally, the high level of admixture found in Notheastern (Catalonia) and especially in Eastern (Valencia and Balearic Islands) accessions may indicate that higher interchange/flowing of plant material, could have occurred in this area, probably due to human displacement within and outside the peninsula territories (Belaj et al., 2004; Guzman Álvarez, 2007; Tous and Franquet i Bernis, 2019).

This work represents a significant enlargement of the conserved germplasm of cultivated olive in Spain. The fact that, through the surveys conducted here, there was an increase of more than 70% in the national olive germplasm accurately identified, indicates that there may still be endangered minor cultivars yet to be discovered in other olive-producing countries. And that giving the extension of the modern olive growing with very few cultivars, this cryptic germplasm is in great danger of disappearance. In our case, the new cultivars identified showed a high level of genetic diversity among and within Spanish regions, locating a new hot spot of diversity in northern regions of the country. Some of the minor cultivars recovered were well adapted to particular environmental conditions and could harbour agronomical traits with a great potential for future national olive breeding programs aimed to mitigate climate change impact on the country. Also, local cultivars could be a very useful source of genes with great potential against new and unforeseen biotic and abiotic stresses, outburst of new pests and diseases, like the case of *Xylella fastidiosa*, as well as for improving oil quality or adapting to new market trends. In addition, the broadening of the collection may play an important role in enlarging the knowledge about olive genetic structure and relationships, which may be of interest in future genome-wide association studies and genitors selection in olive breeding programs. The set of 96 EST-SNPs markers here used proved to be an efficient tool for the identification and recovery of those minor endangered cultivars and is available to those researchers willing to perform similar works in other olive growing countries.

## Data availability statement

The raw data supporting the conclusions of this article will be made available by the authors, without undue reservation.

## Author contributions

FG-G: Data curation, Formal analysis, Methodology, Resources, Software, Visualization, Writing – original draft. AN: Data curation, Resources, Validation, Visualization, Writing – review & editing. JR: Resources, Writing – review & editing. SC: Resources, Writing – review & editing. JA: Resources, Writing – review & editing. JR: Resources, Writing – review & editing. IA: Resources, Writing – review & editing. JV-A: Resources, Writing – review & editing. JC-G: Resources, Writing – review & editing. ZS: Data curation, Formal analysis, Methodology, Software, Writing – review & editing. IL: Data curation, Formal analysis, Software, Writing – review & editing. RR-N: Conceptualization, Resources, Formal analysis, Funding acquisition, Methodology, Validation, Writing – review & editing, Writing – original draft. AB: Conceptualization, Formal analysis, Funding acquisition, Investigation, Methodology, Project administration, Resources, Supervision, Validation, Writing – review & editing.
