# Supplementary material for: New insights in the Spanish gene pool of olive (Olea europaea L.) preserved ex situ and in situ based on high-throughput molecular markers
Source: Front Plant Sci. 2024 Jan 5;14:1267601. doi: 10.3389/fpls.2023.1267601 (PMC10796691; doi:10.3389/fpls.2023.1267601)
Supplement: Supplementary file 1 [file DataSheet_1.zip › Supplementary figures.docx]

Supplementary Material

# Supplementary Figures and Tables

## Supplementary Tables

**Supplementary Table 1**. List of different Spanish cultivars used for diversity and structure analysis. The proportion of genome assigned to the clusters (K=2 and K=3) defined with the model-based clustering method and the Linear Discriminants (LD) defined with the DAPC method are shown**.**

**Supplementary Table 2**. Homonymies detected in this study.

**Supplementary Table 3**. Diversity parameters of the 96 EST-SNPs genotyped in the 427 distinct genotypes.

## Supplementary Figures

**Supplementary Figure 1.** Log-likelihood values for the data conditional of K, ln Pr(X|K), as suggested by Pritchard et al. (2000), and on ΔK values.

**
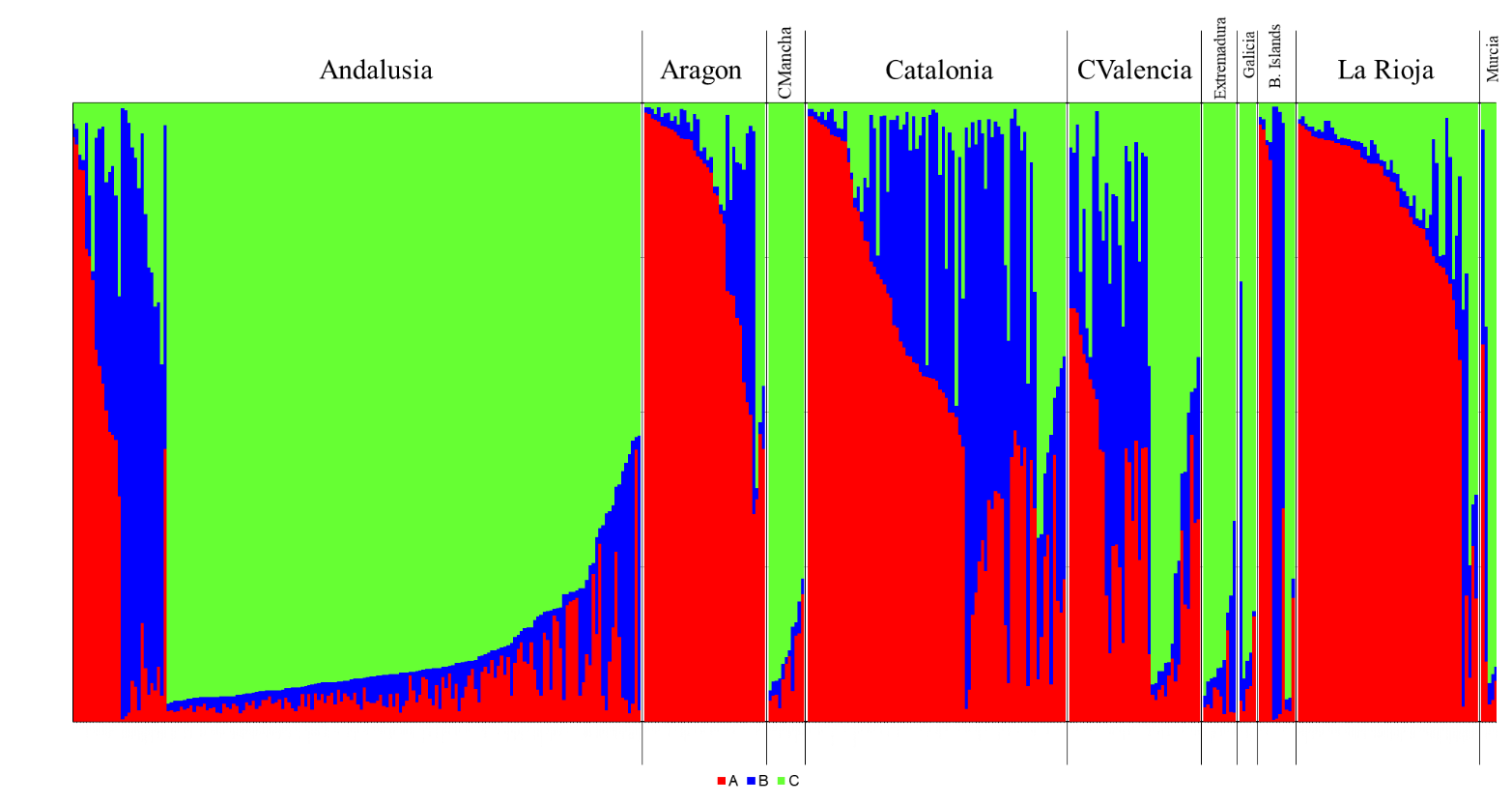
**

**Supplementary Figure 2.** Bar plot of the membership coefficient for k= 3 as calculated by STRUCUTRE ordered by region. Each cultivar is represented by a single vertical line divided intocolours. Each colour represents one cluster, and the length of the coloured segment shows the individual’s estimated proportion of membership in that cluster.


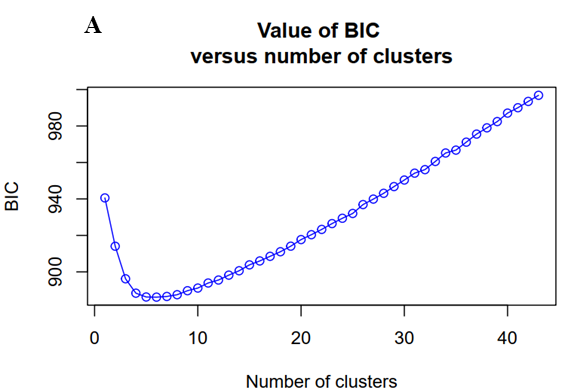

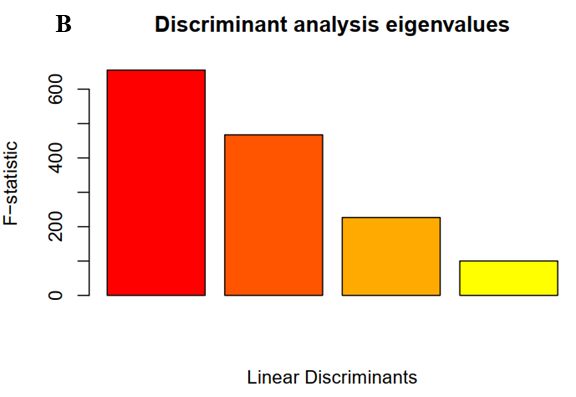


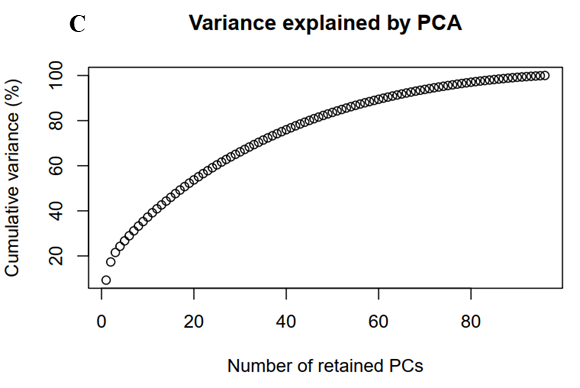


**Supplementary Figure 3.** Plots for the selection of the optimal number of clusters using the lowest Bayesian Information Criterion (A), Eigenvalues of retained discriminant functions (B), and cumulative variance explained by the principal component analysis (PCA) relative to the number of principal components (PCs) retained in the analysis (C).
